# Domestic Animals and Human Infection

**Published:** 1920-08-28

**Authors:** 


### DOMESTIC ANIMALS AND HUMAN INFECTION.

In popular medical literature there will always be some occasional references to the dangers of human infection arising from the diseases to which domestic animals are liable, and it is perhaps not surprising that every now and then a scare upon the subject arises. Although it is more than possible that the real frequency with which children are exposed to such risks tends to be exaggerated, yet it cannot be too strongly impressed upon the general public that the bodies of animals, in either the acute or chronically diseased conditions or in the capacity apparently of healthy carriers, can and frequently do harbour germs of high pathogenic power towards the human race.

At the recent Birmingham Congress of the Royal Sanitary Institute, Dr. Savage, the county M.O.H. for Somerset, dealt with three specific instances, namely, inflammatory conditions of the udders of cows, as they affect the milk of these animals, animals as direct carriers of "food-poisoning" bacteria, and the liability of domestic cats to diphtheria. In connection with the first two, his findings, as regards danger, are distinctly positive, but fortunately the reverse holds good with the last.

Leaving aside the well-known fact that tubercle bacilli can be and undoubtedly are transferred to milk from tuberculous conditions of the bovine mammary glands, it is a point to be remembered that, however well the tuberculin test is applied and milk supervised from this point of view, yet extensive and serious outbreaks of septic sore-throat and its complications can undoubtedly arise, from the presence of hæmolytic streptococci gaining entrance to the milk from a transient streptococcal mastitis in the cow. Ultimately this condition is easily recognised in the bovine, but when we learn that, on tracing the origin of the infection from the cow's point of view, it is found in the great majority of cases that it comes from contaminated hands of the milker, yet another reason should be appreciated for care and cleanliness. Otherwise the first blame may be easily laid at the wrong door.

Also the true cause of contamination of food by pathogenic bacteria may not in any sense lie with the animal itself. We must remember that all animal foods form ideal culture media for bacteria, as Dr. Priestley explains in his investigation of a local outbreak (THE HOSPITAL, August 21, p. 524), and although the animal or its secretions *may* cause the contamination, yet far more frequently this occurs through the agency of rats, mice, or other vermin, or the handling of the food by a human carrier.

With regard to the diphtheria danger coming from pet cats, a generally accepted and, indeed, a text-book view, Dr. Savage's findings are distinctly interesting. He isolated a few diphtheroid organisms from the throats of healthy and catarrhal cats, but in no case was the true Klebs-Löffler bacillus found. Even when the cats were closely associated with known cases of human diphtheria the same negative results were obtained, and nothing suggesting any common occurrence of feline diphtheria-carriers was noticed, even after the most careful bacteriological investigation. More conclusive still is the fact that Dr. Savage, after many attempts, completely failed actively to inoculate virulent human diphtheria bacilli on to the throats of either kittens or grown cats. The bacilli could never afterwards be recovered from the animals and nothing approaching a carrier condition was ever produced.

Nevertheless the public must never forget what an intimate part is played by many animals in the transference, or the life cycles, of disease-producing organisms. The more experimental work, too, that is done on the possibilities among the domestic varieties the better.

